# Do Children With Attention-Deficit/Hyperactivity Disorder Symptoms Become Socially Isolated? Longitudinal Within-Person Associations in a Nationally Representative Cohort

**DOI:** 10.1016/j.jaacop.2023.02.001

**Published:** 2023-06

**Authors:** Katherine N. Thompson, Jessica C. Agnew-Blais, Andrea G. Allegrini, Bridget T. Bryan, Andrea Danese, Candice L. Odgers, Timothy Matthews, Louise Arseneault

**Affiliations:** a King’s College London, London, United Kingdom; b Queen Mary University London, London, United Kingdom; c University College London, London, United Kingdom; d South London and Maudsley NHS Foundation Trust, London, United Kingdom; e University of California Irvine, California, and Duke University, Durham, North Carolina

**Keywords:** ADHD, bidirectional, informants, longitudinal, social isolation

## Abstract

**Objective:**

This study examined longitudinal associations between attention-deficit/hyperactivity disorder (ADHD) symptoms and social isolation across childhood. The study tested the direction of this association across time, while accounting for preexisting characteristics, and assessed whether this association varied by ADHD presentation, informant, sex, and socioeconomic status.

**Method:**

Participants included 2,232 children from the Environmental Risk (E-Risk) Longitudinal Twin Study. ADHD symptoms and social isolation were measured at ages 5, 7, 10, and 12. Random-intercept cross-lagged panel models were used to assess the directionality of the association across childhood.

**Results:**

Children with increased ADHD symptoms were consistently at increased risk of becoming socially isolated later in childhood, over and above stable characteristics (β = .05-.08). These longitudinal associations were not bidirectional; isolated children were not at risk of worsening ADHD symptoms later on. Children with hyperactive ADHD presentation were more likely to become isolated, compared with inattentive presentation. This was evident in the school setting, as observed by teachers, but not by mothers at home.

**Conclusion:**

The study findings highlight the importance of enhancing peer social support and inclusion for children with ADHD, particularly in school settings. This study adds explanatory value beyond traditional longitudinal methods, as the results represent how individual children change over time, relative to their own preexisting characteristics.

**Diversity & Inclusion Statement:**

We worked to ensure sex and gender balance in the recruitment of human participants. We worked to ensure that the study questionnaires were prepared in an inclusive way. One or more of the authors of this paper self-identifies as a member of one or more historically underrepresented sexual and/or gender groups in science. We actively worked to promote sex and gender balance in our author group. The author list of this paper includes contributors from the location and/or community where the research was conducted who participated in the data collection, design, analysis, and/or interpretation of the work.

Social isolation in childhood can be detrimental to physical and mental health.^1^^,^^2^ Children with neurodevelopmental disorders, such as attention-deficit/hyperactivity disorder (ADHD), may be particularly at risk for becoming socially isolated. ADHD is characterized by inattention and/or hyperactivity/impulsivity that interferes with daily functioning.^3^ Children with ADHD often report difficulties in establishing friendships.^4^, ^5^, ^6^ These hyperactive/impulsive and inattentive symptoms influence how children talk to each other, register social cues, and cooperate,^7^^,^^8^ increasing their risk of estrangement from other children.^9^

ADHD symptoms have been shown to increase the likelihood of later social isolation in childhood, and by becoming more isolated over time, children may show an increase in ADHD symptoms over and above prior symptoms.^10^^,^^11^ Isolated children have limited opportunities to observe, model, and learn age-appropriate interpersonal interactions with other children.^12^ Over time, this could increase behaviors such as interrupting conversations, difficulty taking turns, and not paying attention to details. Therefore, children at risk for, or experiencing, ADHD symptoms could become socially isolated, which in turn may worsen their hyperactive/impulsive and inattentive symptoms, creating a negative reinforcement loop in which it is more difficult to form friendships.^7^ Longitudinal modeling techniques can disentangle these potential reciprocal associations and accurately reflect predictions in how children change over time.

The extent to which children with ADHD experience social isolation may vary according to several factors. First, isolation could manifest differently depending on ADHD presentation. Inattentive children withdraw or disengage from social situations, missing key observational learning,^9^ whereas hyperactive/impulsive children could evoke negative responses from people around them.^13^ School-based research shows that children exclude their hyperactive peers from academic and social group activities.^14^ As children get older, those with disruptive or impulsive behaviors that do not conform to typical social norms could be increasingly rejected by peers.^15^ Second, the context in which children interact with peers could play a role in what behavior is observed and by whom. For example, schools provide structured social environments in which teachers monitor groups of children of the same age and have a wide reference to compare children’s behavior and a context in which children with ADHD may be excluded by peers because of their symptoms. In contrast, at home ADHD symptoms may be shared within the family and accepted. ADHD symptoms could therefore be more distinguishable to teachers than to parents. This is supported by consistently modest agreement between parents and teachers on child behavior,^16^ and both perspectives should be accounted for when assessing peer relationships and ADHD.^17^ Third, prevalence rates of ADHD and social isolation can differ substantially by sex and socioeconomic status (SES). Boys are more likely than girls to experience and be diagnosed with severe symptoms of ADHD.^18^ Similarly, low SES is associated with many types of adversity,^19^ and social isolation has shown to be frequent in disadvantaged socioeconomic groups.^20^

Key social behaviors involved in building positive or negative interactions should be accounted for to ensure the independence of this association and to better understand the pathway by which children with ADHD symptoms become isolated. Negative outward behavior toward other children may account for the association between ADHD symptoms and social isolation. ADHD in childhood often co-occurs with antisocial, disruptive, or aggressive behaviors.^21^ These behaviors could evoke long-term negative responses from others in a child’s environment. Previous research showed that childhood ADHD symptoms accompanied by high rates of aggression were associated with social problems years later.^15^ Similarly, longitudinal work demonstrated that childhood ADHD when comorbid with conduct disorder was associated with later neglect.^13^ This potential mediating effect of antisocial behaviors could differ by ADHD presentation; hyperactive/impulsive rather than inattentive symptoms may drive associations with social isolation.^14^^,^^21^ Alternatively, children with ADHD symptoms who have developed prosocial behaviors could be protected from being isolated.^22^

In the current study, we investigated the associations between ADHD symptoms and social isolation across childhood in a nationally representative longitudinal cohort in the United Kingdom. First, we aimed to test the direction of the associations between ADHD and isolation while accounting for preexisting characteristics. We hypothesized that there would be bidirectional associations between experiences of social isolation and ADHD symptoms across childhood. Second, we aimed to ensure that the association between ADHD symptoms and social isolation cannot be explained by co-occurring antisocial or prosocial behaviors. We hypothesized that antisocial or prosocial behaviours would mediate the association between social isolation and ADHD symptoms. Third, we aimed to assess if the association differed for hyperactive/impulsive and inattentive presentations. We hypothesized that the association with social isolation would be stronger for children with a hyperactive presentation of ADHD symptoms. Fourth, we aimed to test for moderating effects of informant (parents and teachers), sex, and SES. We hypothesized that there would be differences in the strength of the association when stratified by informant, sex, and SES.

## Method

### Participants

Participants were members of the Environmental Risk (E-Risk) Longitudinal Twin Study, which tracks the development of 2,232 British children. The sample was drawn from a larger birth cohort of twins born in England and Wales in 1994-1995.^23^ Full details about the sample are reported elsewhere.^24^ Briefly, E-Risk was constructed in 1999-2000, when 1,116 families (93% of those eligible) with same-sex 5-year-old twins participated in home-visit assessments. This sample comprised 56% monozygotic and 44% dizygotic twin pairs; sex was evenly distributed within zygosity (49% male participants); 90% of participants were of White ethnicity. The sample reflects the variety of socioeconomic conditions in the United Kingdom, as reflected in the families’ distribution on neighborhood-level socioeconomic indices (Table 1).^25^ Groups of low, middle, and high SES were derived based on household income and highest education qualification. Follow-up home visits were conducted when children were 7 (98% participation), 10 (96% participation), 12 (96% participation), and 18 (93% participation) years of age. Visits at ages 5-12 included assessments with participants and their mother (primary caretaker). Sample characteristics are included in Supplement 1, Figure S1, and Tables S1 and S2, available online. The Joint South London and Maudsley and the Institute of Psychiatry Research Ethics Committee approved each phase of the study. Parents gave informed consent, and participants between 5 and 12 years of age gave assent.

**Table 1:** Key Characteristics of the Environmental Risk Longitudinal Twin Study Measured at Age 5

	Male participants (n = 1,092)		Female participants (n = 1,140)		Total participants (N = 2,232)	
Social isolation^a^						
Mean (SD)	0.88 (1.23)		0.75 (1.03)		0.81 (1.13)	
Minimum	0		0		0	
Maximum	12		9		12	
ADHD symptoms^a^						
Mean (SD)	2.67 (3.01)		1.85 (2.45)		2.25 (2.76)	
Minimum	0		0		0	
Maximum	16		18		18	

	n	(%)	n	(%)	n	(%)
Race						
Asian	36	(1.61)	54	(2.42)	90	(4.03)
Black	18	(0.81)	24	(1.08)	42	(1.89)
Mixed race	4	(0.18)	4	(0.18)	8	(0.36)
Not listed	36	(1.61)	38	(1.70)	74	(3.31)
White	998	(44.71)	1,020	(45.70)	2,018	(90.41)
Family SES						
Low	360	(16.13)	382	(17.11)	742	(33.24)
Middle	370	(16.58)	368	(16.49)	738	(33.07)
High	362	(16.22)	390	(17.47)	752	(33.69)
Neighborhood SES^b^						
Wealthy achievers	250	(11.20)	248	(11.11)	498	(22.31)
Urban prosperity	60	(2.69)	64	(2.87)	124	(5.56)
Comfortably off	294	(13.17)	322	(14.43)	616	(27.60)
Moderate means	158	(7.08)	166	(7.44)	324	(14.52)
Hard pressed	310	(13.89)	316	(14.16)	626	(28.05)
Unknown	20	(0.90)	24	(1.08)	44	(1.98)

Note: ADHD = attention-deficit/hyperactivity disorder; SES = socioeconomic status.

a Combined mother and teacher reports at age 5.

b ACORN Index at age 5 (CACI Ltd., London).

### Measures

#### Social Isolation and ADHD Symptoms

At ages 5, 7, 10, and 12, social isolation and ADHD symptoms were assessed using items from the Child Behavior Checklist (CBCL)^26^ and matching items from the Teacher’s Report Form (TRF).^27^ Social isolation items included the following: “would rather be alone than with others,” “not liked by other children,” “doesn’t get along with other children,” “feels or complains that no-one loves him/her,” “withdrawn, doesn’t get involved with others,” and “complains of loneliness.” This approach maps onto the working definition of childhood social isolation by Caspi *et al.*,^1^ which is conceptualized as lack of social relationships through social rejection or withdrawal from other children. ADHD items reflected 9 symptoms of inattention and 9 symptoms of hyperactivity/impulsivity based on *DSM-IV*^28^ (Supplement 2 and Table S3, available online). Mothers completed the questionnaire in a face-to-face interview at each age, and teachers responded to the same items by mail. All item responses were scored 0 (“not true”), 1 (“somewhat true”), and 2 (“often true”). ADHD symptoms were considered present only if “often true” was endorsed.

Items were summed to create separate mother and teacher scales for social isolation and ADHD at each age (social isolation reporter *r*s at each age = 0.26-0.31; ADHD *r*s = 0.26-0.35). We averaged mothers’ and teachers’ reports to form a combined scale that integrates observations from different settings. Apart from analyses assessing informant differences, all models used the combined score (averaged reports from mothers and teachers). For ADHD, scores were summed separately for inattention and hyperactivity/impulsivity, then combined to create a total ADHD score. A total of 2,078 (93.10%) children had complete social isolation and ADHD data at all 4 time points. Missingness was handled using maximum likelihood estimation; thus all children had at least one rating of ADHD symptoms and social isolation and were included in analyses (N = 2,232).

#### Prosocial and Antisocial Behaviors

Antisocial and prosocial behaviors were assessed at ages 5, 7, 10, and 12. Prosocial behavior included 10 items taken from the revised Rutter parent questionnaire^29^ that reflect actions beneficial to others (for example, “considerate of other people’s feelings” or “tries to help someone who has been hurt”). Antisocial behavior was assessed using the CBCL and TRF consisting of items that represent subscales of aggression (eg, “bullying or threatening people”), delinquency (eg, “steals outside the home”), and conduct problems (eg, “annoys people on purpose”). Responses were scored as described above and summed to create mother and teacher scores at each age (prosocial *r*s at each age = 0.13-0.19; antisocial *r*s = 0.30-0.40). See Table S4, available online, for the full list of items.

### Statistical Analyses

#### Associations Between Social Isolation and ADHD Across Childhood

We used the random intercept cross-lagged panel model (RI-CLPM)^30^ to assess bidirectional associations between social isolation and ADHD across ages 5, 7, 10, and 12 years. RI-CLPM is a structural equation model that builds on the traditional CLPM by disentangling change that occurs for each person over time (within-person) and change that occurs on average for groups of people over time (between-person). To provide accurate predictive insight for prevention and intervention strategies, it is key to understand how a child’s behavior at one time point can directly impact another behavior later on. These within-person processes cannot be investigated using traditional CLPM, as these are conflated with between-person processes, which may yield spurious results and biased parameter estimates.^31^

RI-CLPM adds random intercepts to the CLPM for each variable, which accounts for stable, traitlike differences between individuals. This separates change that happens within one child from one time point to the next (within-person), while accounting for stable, time-invariant, individual differences (between-person). RI-CLPM is made up of autoregressive paths, which represent change or carryover effects in one construct over time (eg, age 5 to age 7 isolation), and cross-lag paths, which represent within-person bidirectional effects from one variable to the other over time (eg, age 5 ADHD to age 7 social isolation). We refer to within-person associations as individual-level and the combination of within-person and between-person associations as group-level.

We first fitted CLPM, then RI-CLPM as the next step to determine whether the CLPM is confounded by between-person effects. We used the chi-bar-square test to assess the improvement in model fit from CLPM to RI-CLPM. For each model, parameters were allowed to vary freely over time; this model was then compared with a nested model where equality constraints were imposed for autoregressive and cross-lag paths to determine whether the model could be simplified to be consistent over time without reducing model fit. To test if associations were consistent across ADHD symptoms, we computed RI-CLPM for hyperactivity/impulsivity and inattention symptoms separately. To check that the association between ADHD symptoms and social isolation was not due to antisocial or prosocial behaviors, we conducted within-person longitudinal mediation using RI-CLPM. We examined cross-lag paths for potential mediation effects (Figure S2, available online) and indirect paths (a × b) were tested for significance at *p* < .05. As a sensitivity analysis, we assessed the measurement invariance of social isolation and ADHD over time. Evidence of measurement invariance was found for both constructs, supporting the conclusion that the measures captured the same construct over time (Supplement 3 and Table S5, available online).

#### Informant, Sex, and SES Differences

To assess informant discrepancies, separate RI-CLPMs were fit for teacher- and mother-reported ADHD symptoms and social isolation to see if cross-lag path estimates differed depending on informant. Multiple group RI-CLPM^32^ was used to assess sex and SES differences. The similarity of the within-person cross-lag effects was compared by stratifying across different groups (male/female participants; low/medium/high SES) and comparing model fit using χ^2^ difference tests. To do this, autoregressive and cross-lag paths were constrained to be equal across groups and then allowed to be freely estimated; the model was assessed for a substantial decrease in fit. These represent moderation, or interaction, effects whereby group membership influences the strength or direction of the longitudinal association between social isolation and ADHD.

Model fit was evaluated by examining the comparative fit index (CFI; >0.95 for adequate fit), Tucker-Lewis index (TLI; >0.95), root mean square error of approximation (RMSEA; <0.06), and standardized root mean square residual (SRMR; <0.08). Maximum likelihood estimation with robust test statistics and robust standard errors was used throughout all analyses to account for nonnormality and nonindependence of twin pairs. The Satorra-Bentler scaled χ^2^ difference test was used for all χ^2^ difference testing. All models were estimated in R v4.0.3 (R Foundation for Statistical Computing, Vienna, Austria) using lavaan (v0.6-10).^33^ The analysis plan was preregistered (https://sites.duke.edu/moffittcaspiprojects/files/2022/02/ThompsonK_2022_bidirectional-associations_Erisk.pdf), and code is on GitHub (https://knthompson26.github.io/social-isolation-ADHD-bidirectional-effects/).

## Results

### Do Social Isolation and ADHD Symptoms Predict Each Other Across Childhood?

#### Group-Level Associations

**Figure 1:**
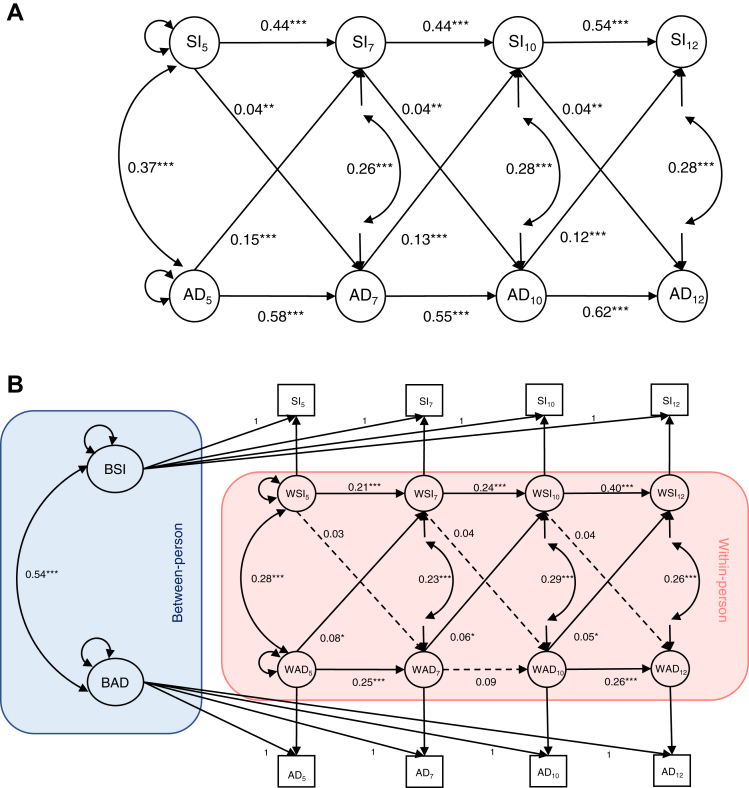
Longitudinal Association Between Social Isolation and Attention-Deficit/Hyperactivity Disorder (ADHD) Symptoms Across Ages 5, 7, 10, and 12 Using Cross-Lagged Panel Model (A) and Random-Intercept Cross-Lagged Panel Model (B) ***Note:****Nonsignificant regression paths are indicated by dashed one-headed arrows. Significant regression paths are indicated by solid one-headed arrows. Correlation paths are indicated by double-headed arrows. Subscript numbers indicate time point of assessment. Combined mother and teacher scores were used at all time points. Within-person level of the random-intercept cross-lagged panel model (B) is indicated in pink, and between-person is indicated in blue. Cross-lag paths were constrained to be equal across time. AD = measured ADHD symptom sum score; BAD = between-person level ADHD represented by a random intercept; BSI = between-person social isolation represented by a random intercept; SI = measured social isolation sum score; WSI = within-person level factor of social isolation; WAD = within-person level factor of ADHD.* *∗*p *< .05; ∗∗*p *< .01; ∗∗∗*p *< .001.*

Social isolation and ADHD symptoms were relatively stable from one time point to the next and concurrently associated with one another throughout childhood according to the CLPM (Figure 1A). On average, higher ADHD symptoms were associated with later increasing levels of social isolation, and, to a lesser extent, higher levels of social isolation were associated with increasing ADHD symptoms later on. These group-level bidirectional associations were consistent throughout childhood (Supplement 4 and Table S6, available online).

#### Individual-Level Associations

By partialing out group- and individual-level associations using RI-CLPM, the stable group-level between-person associations showed that, on average, children who were more isolated also experienced increased ADHD symptoms (*r* = 0.54, *p* < .001) (Tables S7-S9, available online). The RI-CLPM fit the data substantially better than the CLPM (χbar^2^diff = 337.52, *p* < .001) (Table 2). The most parsimonious RI-CLPMs consisted of constrained cross-lag paths for the combined and mother-report models and constrained autoregressive and cross-lag paths for the teacher-report model (Supplement 5 and Tables S10-S12, available online). The individual-level autoregressive effects in the RI-CLPM showed that a fluctuating increase in social isolation at one age predicted increases in social isolation at the subsequent time point, with similar but slightly less consistent autoregressive effects for ADHD symptoms (Figure 1B). When accounting for preexisting stable differences between children (the random intercepts), the association between social isolation and ADHD symptoms differed from that obtained using CLPM; cross-lag effects showed that an increase in ADHD symptoms (relative to each individual’s average level of symptoms) was associated with increased levels of social isolation later on (Figure 1B). However, increased levels of social isolation did not predict a later increase in ADHD symptoms. These effects were consistent across childhood (Supplement 5, available online), and longitudinal mediation analyses showed that this was not mediated by antisocial or prosocial behaviors (Supplement 6, Figure S3, and Table S13, available online).

**Table 2:** Cross-Lagged Panel Model (CLPM) and Random-Intercept Cross-Lagged Panel Model (RI-CLPM) Fit Statistics for Combined Attention-Deficit/Hyperactivity Disorder (ADHD) Symptoms, Hyperactivity, and Inattention

	Scaled χ^2^	*df*	CFI >0.95	TLI >0.95	Adjusted BIC	RMSEA <0.06	RMSEA 95% CI	SRMR <0.08
ADHD								
CLPM	225.03	16	0.94	0.89	63900.15	0.10	0.09, 0.12	0.07
RI-CLPM	43.18	13	0.99	0.98	63575.22	0.04	0.02, 0.06	0.03
Hyperactivity								
CLPM	218.26	16	0.94	0.89	54690.29	0.10	0.09, 0.11	0.06
RI-CLPM	35.93	13	0.99	0.99	54387.60	0.04	0.02, 0.05	0.03
Inattention								
CLPM	232.21	16	0.93	0.88	53371.98	0.11	0.09, 0.12	0.07
RI-CLPM	40.02	13	0.99	0.98	53025.07	0.04	0.03, 0.06	0.03

Note: The cutoff to signify good fit is given in each header.^52^ All fit statistics reported are robust to account for nonnormality and paired twin data. All models have cross-lag paths constrained to be equal across time. ADHD = attention-deficit/hyperactivity disorder; BIC = bayesian information criterion; CFI = comparative fit index; CLPM = cross-lagged panel model; RI-CLPM = random-intercept cross-lagged panel model; RMSEA = root mean square error of approximation; SRMR = standardized root mean square residual; TLI = Tucker-Lewis index.

### Do Associations With Social Isolation Differ According to ADHD Presentation?

**Figure 2:**
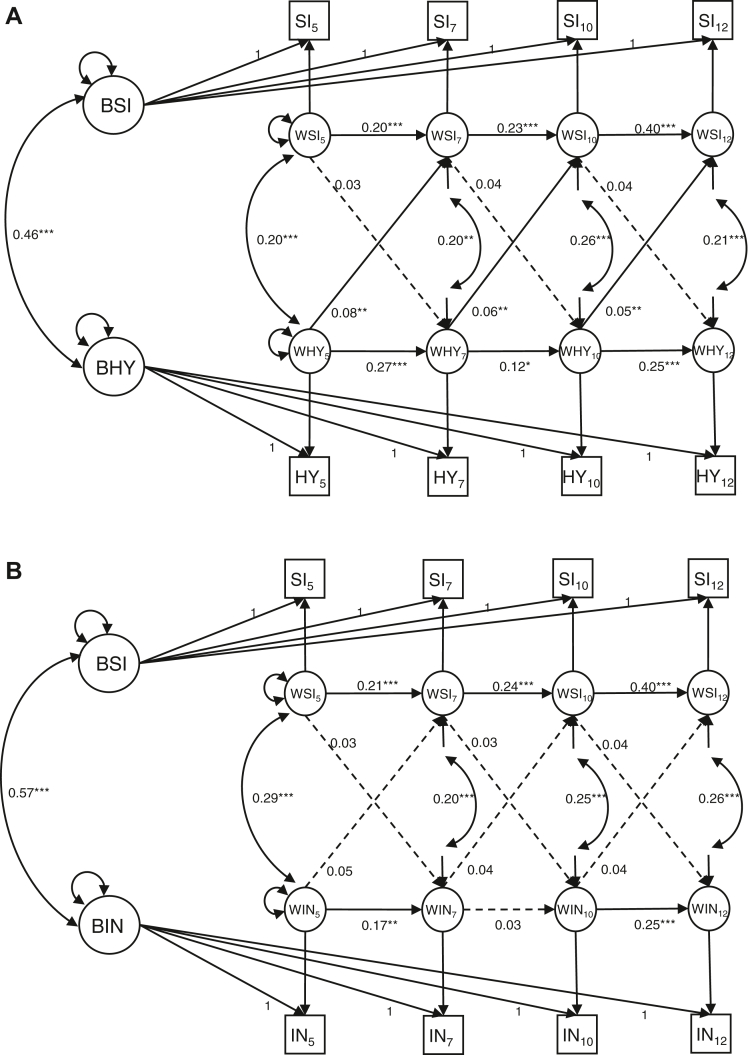
Longitudinal Associations With Social Isolation According to Different Attention-Deficit/Hyperactivity Disorder Presentations ***Note:****(A) The random-intercept cross-lagged panel model representing the longitudinal association between social isolation and hyperactivity across ages 5, 7, 10, and 12. (B) The random-intercept cross-lagged panel model representing the longitudinal association between social isolation and inattention across ages 5, 7, 10, and 12. Nonsignificant regression paths are indicated by dashed one-headed arrows. Significant regression paths are indicated by solid one-headed arrows. Correlation paths are indicated by double-headed arrows. Subscript numbers indicate time point of assessment. Combined mother and teacher scores were used at all time points. Both models have cross-lag paths constrained to be equal across time. BHY = between-person level hyperactivity represented by a random intercept; BIN = between-person level inattention represented by a random intercept; BSI = between-person social isolation represented by a random intercept; HY = measured hyperactivity symptom sum score; IN = measured inattention symptom sum score; SI = measured social isolation sum score; WHY = within-person level factor of hyperactivity; WIN = within-person level factor of inattention; WSI = within-person level factor of social isolation.* *∗*p *< .05; ∗∗*p *< .01; ∗∗∗*p *< .001.*

Separate models for symptoms of hyperactivity/impulsivity and inattention both showed good fit to the data using RI-CLPM (Table 2). The between-person or group-level associations were consistent with the combined ADHD symptoms model; children who experienced increased inattention or hyperactivity/impulsivity symptoms, on average, were also more isolated (*r* = 0.57, *r* = 0.46). At the individual level, cross-lag paths differed for hyperactivity/impulsivity and inattention: similar to the combined ADHD symptom model, we found significant cross-lag effects from hyperactivity to later social isolation (Figure 2A). However, no such pattern was observed for inattention (Figure 2B). Across both models, as with combined ADHD, the cross-lag effect of social isolation on later inattentive and hyperactive symptoms was nonsignificant.

### Do Associations Between Childhood Social Isolation and ADHD Symptoms Differ According to Informants, Sex, and SES?

Associations between ADHD symptoms and later social isolation were significant for teacher reports, but not for mother reports (Table 3). When stratified by ADHD symptoms, we found this effect for teacher-reported hyperactivity (β = .074-.105, *p* = .001), but not inattention (β = .046-.058, *p* = .091). We found no overall sex differences for combined ADHD symptoms or for hyperactive/impulsive or inattentive presentations (Table 3). Although not significantly different overall, the effect of increased ADHD symptoms on later social isolation was more pronounced in boys. There were differences according to family SES: the longitudinal paths from ADHD to social isolation were significant for middle, but not low and high, SES groups. However, when computed separately for each informant, all global SES group differences became nonsignificant (*p* = .303-.612), and only longitudinal paths for low SES remained significant. This suggests that these apparent SES differences (Table 3) are driven and confounded by combining informant reports (details provided in Tables S14-S16, available online).

**Table 3:** Random-Intercept Cross-Lagged Panel Model (RI-CLPM) Cross-Lag Effect Estimates Stratified by Sex, Socioeconomic Status (SES), and Informant (Mother and Teacher)

	ADHD predicting isolation (standardized β)			Isolation predicting ADHD (standardized β)			Chi-square difference test
	Age 5-7	Age 7-10	Age 10-12	Age 5-7	Age 7-10	Age 10-12	
ADHD informant differences							
Mother	0.063†	0.047†	0.040†	0.031	0.037	0.041	
Teacher	0.091∗	0.078∗	0.067∗	−0.026	−0.032	−0.040	
Hyperactivity informant differences							
Mother	0.059	0.045	0.035	0.030	0.039	0.045	
Teacher	0.105∗∗	0.089∗∗	0.074∗∗	−0.004	−0.005	−0.006	
Inattention informant differences							
Mother	0.055	0.041	0.040	0.031	0.033	0.036	
Teacher	0.058	0.051	0.046	−0.032	−0.038	−0.048	
ADHD sex differences							χ^2^Δ = 6.76, dfΔ = 8, *p* = .563
Female participants	0.063	0.041	0.037	−0.003	−0.004	−0.004	
Male participants	0.090†	0.074†	0.062†	0.062	0.067	0.074	
Hyperactivity sex differences							χ^2^Δ = 9.83, dfΔ = 8, *p* = .277
Female participants	0.050	0.032	0.028	0.003	0.004	0.004	
Male participants	0.096∗	0.079∗	0.062∗	0.049	0.058	0.067	
Inattention sex differences							χ^2^Δ = 4.22, dfΔ = 8, *p* = .837
Female participants	0.060	0.041	0.037	0.008	0.009	0.011	
Male participants	0.052	0.042	0.041	0.055	0.052	0.057	
ADHD SES differences							χ^2^Δ = 35.80, dfΔ = 16, *p* = .003
Low	0.123∗	0.100∗	0.077∗	0.071	0.072	0.072	
Middle	0.169∗∗∗	0.126∗∗∗	0.107∗∗∗	−0.023	−0.029	−0.033	
High	−0.065	−0.043	−0.050	0.027	0.029	0.039	
Hyperactivity SES differences							χ^2^Δ = 35.58, dfΔ = 16, *p* = .003
Low	0.089	0.070	0.056	0.065	0.064	0.065	
Middle	0.182∗∗∗	0.146∗∗∗	0.111∗∗∗	−0.017	−0.023	−0.027	
High	−0.035	−0.023	−0.021	0.027	0.036	0.045	
Inattention SES differences							χ^2^Δ = 30.99, dfΔ = 16, *p* = .013
Low	0.090	0.070	0.056	0.065	0.064	0.065	
Middle	0.182∗∗∗	0.146∗∗∗	0.112∗∗∗	−0.017	−0.023	−0.027	
High	−0.035	−0.023	−0.020	0.027	0.036	0.045	

Note: Sex and SES models use combined (averaged) reports from parents and teachers. Robust χ^2^ used in all models. The χ^2^ difference test is not applicable for reporter differences, as model was created from separate data (not nested). Combined report and mother report include constrained cross-lag effects. Teacher report models include constrained autoregressive and cross-lag effects. ADHD = attention-deficit/hyperactivity disorder; SES = socioeconomic status.

∗ *p* < .05; ∗∗*p* < .01; ∗∗∗*p* < .001; †*p* = .05.

## Discussion

Our work provides new insight into longitudinal associations between social isolation and ADHD symptoms across childhood. Although research suggests a link between ADHD and social exclusion,^4^^,^^7^ no studies have established the direction of this association or disentangled the influence of stable traits and fluctuating behavior. We found that children with increased ADHD symptoms were at risk of becoming socially isolated later in childhood, and this was most evident when reported by teachers or for hyperactive children in the school setting. Altogether, our findings point toward the role of neurodevelopmental disorders in shaping social experiences later in life.

A focus on how behavior changes, rather than trait differences, is key for this study, as social isolation has been shown to be dynamic throughout childhood.^10^ Using traditional methods (CLPM), we showed bidirectional associations between social isolation and ADHD symptoms. However, when using methods that account for stable characteristics (RI-CLPM), evidence indicates that increases in ADHD symptoms lead to increases in social isolation across childhood. CLPM findings can be misleading, as they report overinflated associations that do not accurately represent how individual children change throughout development. Our findings add explanatory value beyond traditional longitudinal models, as they represent how individual children change over time, relative to their own preexisting characteristics. RI-CLPM separates out stable fundamental differences between individuals from within-person processes that may lead from change in one behavior to change in another.^32^ Similar within-person associations have been found for how peer socialization relates to autistic traits and depression^34^^,^^35^ and is comparable to work on individual- vs population-level predictions in child behavior.^36^ Our findings highlight that group-level associations do not capture active behavioral processes underlying why children become more or less socially isolated over time.

We found that a large proportion of the association between ADHD symptoms and social isolation was explained by shared time-invariant traits that make children more likely both to be isolated and to have higher ADHD symptoms. These factors could include social difficulties,^37^ genetic vulnerabilities,^38^ or personality.^39^ When accounting for this confounding of stable traits, we demonstrated that increases in ADHD symptoms directly and consistently lead to more social isolation throughout childhood. We further showed that this was not explained by outward antisocial or prosocial behavior. Rather, it could be that children with ADHD become excluded because of negative actions and perceptions held by their peers. Hyperactive children are often perceived negatively by classmates,^9^ and their maladaptive behaviors could put them in the spotlight for dysfunctional peer interactions.^40^ Research shows that children with ADHD are more likely to be victimized compared with typically developing children.^41^ Negative interactions can increase the feeling of being misunderstood,^7^ and bullying victimization, particularly at school, could lead children with ADHD to become withdrawn, rejected, lonely, and isolated.^42^

When considering separate presentations of ADHD, associations with social isolation were strongest for hyperactive/impulsive symptoms. Children with higher hyperactivity/impulsivity may be left out of peer interactions, as they are more likely to dominate conversations, interrupt, go off on a tangent, and speak only about topics of their interest.^43^ Hyperactive/impulsive, but not inattentive, symptoms may evoke negative responses from others. Children with high levels of hyperactive/impulsive symptoms could be singled out as disruptive and nonconforming to school rules, which may affect how they are perceived by other peers in the classroom.^14^ This is consistent with our finding that the association between ADHD symptoms and social isolation was apparent only to teachers in the school setting. Parents may rate children differently than teachers due to biases in reporting as well as observing their child in distinct settings or contexts. Teachers witness children in a structured social environment designed to promote stillness and quietness. They also observe many interactions between children that would not necessarily occur at home, thus providing a wider frame of reference to observe peer relationships and recognize social isolation. At home, parents of children with ADHD may normalize hyperactive/impulsive symptoms and make an effort to facilitate social interactions with other children.^44^ Our findings emphasize differences in whether, and how, children with ADHD experience social isolation across the contexts of home and school.

Several limitations were noted in this study. First, it is unclear how our findings apply to clinical populations of children with ADHD where more severe presentations of ADHD may be represented in greater numbers. Instead, we show that hyperactive/impulsive symptoms are associated with social isolation even in the general population. Second, models using reports from one informant only could have inflated the associations. We repeated the analyses using different combinations of informants, which showed that regardless of the ADHD informant, teacher reports of isolation were driving the association. This finding is supported by research that validated teacher reports using objective measures of social connectedness in children.^45^ Third, we did not account for confounders in RI-CLPM. Children’s behavior is complex, and it is possible that there are other factors that influenced our findings, for example, bullying victimization. Fourth, our findings from a twin sample may not generalize to singletons. As all participants share a sibling of the same age, this could have inflated our model estimates. One twin’s behavior can reinforce the other twin’s behavior, which makes them more alike over and above stable genetic and environmental factors.^46^ However, twins could still become isolated from their peers despite, and perhaps because of, closeness with their cotwin. For example, children may withdraw or not seek active socialization with peers in favor of the company of their cotwin of the same sex and age.

Our findings have 3 key implications for future research. First, future longitudinal research must distinguish between processes reflecting stable traits and time-sensitive processes. We show that CLPM cannot do this when assessing child behaviour. Disentangling between-person (traitlike similarities) and within-person (temporal fluctuations) associations in psychiatric conditions across childhood is useful to inform individual prediction, understand psychiatric comorbidity, and discover developmental pathways underlying mental health disorders. Second, social isolation could be a mechanism by which children with ADHD may experience increased risk for comorbid mental health disorders. Previous research has shown that limited or harmful relationships can lead to anxiety and depression.^4^^,^^5^ Similarly, an RI-CLPM study showed that social problems precipitated later internalizing problems in childhood.^46^ Research is needed to establish whether social isolation is a catalyst for mental health disorders in children with ADHD, over and above genetic comorbidity. Third, we adopted a broad approach when defining social isolation; future research could focus on different conceptualizations. More work is needed to distinguish the process behind social isolation in children with ADHD, as intervention efforts may benefit from understanding the element of choice in the child being alone. This applies not only to children with ADHD symptoms; future research should assess whether isolation emerges from being rejected by other children or is due to a preference not to engage with other children impact well-being differently.

Our findings also have implications for clinicians and educational professionals. Social difficulties associated with ADHD can be as detrimental as the core symptoms themselves.^47^ Our work suggests that social isolation should be carefully assessed in children with ADHD and that these children could benefit from holistic interventions aimed at easing social challenges. One way to do this could be to incorporate individual-focused intervention. ADHD is highly heterogeneous, and interventions must recognize individual priorities, preferences, and differences in the way children socialize to prevent isolation.^48^ Current social skills interventions for children with ADHD have shown mixed success.^49^ This suggests that children may have the social knowledge but lack the individual tools to feel comfortable in social environments. Another more complex way to improve interventions could be adapting the social environment surrounding children with ADHD. Social impairment experienced by neurodivergent children can be seen as a mismatch between their surroundings and the way they think and behave.^48^ According to young people with ADHD, treatment can heavily focus on medication and less support is provided for managing or establishing peer relationships.^5^ However, medication could potentially ease social challenges through reducing ADHD symptoms themselves.^50^ In conjunction with medical treatment, clinicians could promote modifications that increase social participation in schools and local communities.^51^ In addition to clinical settings, schools can contribute to the comprehensive care for children with ADHD. A shift in adapting the school context to combat negative biases around neurodiversity could provide reductions in social isolation. Advocating inclusive classroom norms in schools has been shown to enhance peer sympathy and inclusion toward hyperactive children.^40^ This could lead to positive peer connections that can be protective against victimization. Teachers can credit the strengths neurodivergent children bring to the classroom and promote positive peer-to-peer interaction and model inclusivity and constructively resolve conflict or reduce deviant attitudes toward hyperactive behavior.^52^

## Supplemental Material

Supplement-Revised R1 Supplement-Revised R1
