# Supplementary material for: Do Children With Attention-Deficit/Hyperactivity Disorder Symptoms Become Socially Isolated? Longitudinal Within-Person Associations in a Nationally Representative Cohort
Source: JAACAP Open. 2023 Feb 21;1(1):12–23. doi: 10.1016/j.jaacop.2023.02.001 (PMC10259183; doi:10.1016/j.jaacop.2023.02.001)
Supplement: Supplement-Revised R1 [file mmc1.docx]

**Do children with ADHD symptoms become socially isolated?**

**Longitudinal within-person associations in a UK representative cohort**

**Supplementary material**

Contents

[Sample characteristics 3](#_Toc129169861)

[Supplement 1 4](#_Toc129169862)

[Socioeconomic description 4](#_Toc129169863)

[Missingness at age 18 4](#_Toc129169864)

[Primary caregiver information 4](#_Toc129169865)

[Missing social isolation data in E-Risk 4](#_Toc129169866)

[Figure S1. Population representativeness of the E-Risk sample 5](#_Toc129169867)

[Table S1: percentage of primary caregivers that completed the “mother report” questionnaire 6](#_Toc129169868)

[Table S2: participants with complete data for social isolation and ADHD symptom sum scores across all time points 6](#_Toc129169869)

[Study items 6](#_Toc129169870)

[Supplement 2 6](#_Toc129169871)

[ADHD diagnosis information 6](#_Toc129169872)

[Table S3. Pairwise correlations between social isolation and ADHD across ages 5, 7, 10, and 12 7](#_Toc129169873)

[Table S4. Full list of items used in the current study 7](#_Toc129169874)

[Conceptual longitudinal mediation 11](#_Toc129169875)

[Figure S2: Longitudinal mediation model using three-variable RI-CLPM 12](#_Toc129169876)

[Longitudinal measurement invariance 12](#_Toc129169877)

[Supplement 3 12](#_Toc129169878)

[Table S5. Measurement invariance testing for RI-CLPM using social isolation and ADHD 12](#_Toc129169879)

[Constraint testing for CLPM 13](#_Toc129169880)

[Supplement 4 13](#_Toc129169881)

[Table S6. Equality constraint testing for combined report CLPM: 𝝌² difference test statistics 13](#_Toc129169882)

[Variances and covariances of random intercepts from RI-CLPM 14](#_Toc129169883)

[Table S7. Variances and covariances of the random intercepts for the total ADHD and social isolation RI-CLPM 14](#_Toc129169884)

[Table S8. Variances and covariances of the random intercepts for the total ADHD, social isolation and antisocial behaviour RI-CLPM 14](#_Toc129169885)

[Table S9. Variances and covariances of the random intercepts for the total ADHD, social isolation and prosocial behaviour RI-CLPM 14](#_Toc129169886)

[Constraint testing for RI-CLPM 15](#_Toc129169887)

[Supplement 5 15](#_Toc129169888)

[Table S10. Equality constraint testing for combined report RI-CLPM: 𝝌² difference test statistics 15](#_Toc129169889)

[Table S11. Equality constraint testing for mother report RI-CLPM: 𝝌² difference test statistics 16](#_Toc129169890)

[Table S12. Equality constraint testing for teacher report RI-CLPM: 𝝌² difference test statistics 17](#_Toc129169891)

[Do prosocial or antisocial behaviours mediate the association between ADHD and isolation? 17](#_Toc129169892)

[Supplement 6 17](#_Toc129169893)

[Figure S3. Longitudinal medication model 19](#_Toc129169894)

[Table S13. Mediation random-intercept cross-lagged panel model for how prosocial and antisocial behaviour mediate the association between ADHD and social isolation. 19](#_Toc129169895)

[Sex and SES differences for mother and teacher report models. 20](#_Toc129169896)

[Table S14. Random-intercept cross-lagged panel model (RI-CLPM) fit statistics for combined ADHD symptoms, hyperactivity, and inattention, split by reporter 20](#_Toc129169897)

[Table S15. Random-intercept cross-lagged panel model (RI-CLPM) cross-lag effect estimates stratified by sex, socioeconomic status (SES), for each informant (mother and teacher) 21](#_Toc129169898)

[Table S16. Iterations of models using different combinations of reporters for isolation and ADHD 23](#_Toc129169899)

[References 24](#_Toc129169900)

# Sample characteristics

## Supplement 1

##

### Socioeconomic description

Families were recruited to represent the UK population with new-borns in the 1990s on the basis of residential location throughout England and Wales and mother’s age. Teenage mothers with twins were over-selected to replace high-risk families selectively lost to the register through non-response. Older mothers having twins via assisted reproduction were under-selected to avoid an excess of well-educated older mothers. The study sample represented the full range of socioeconomic conditions in the UK, as reflected in families’ distribution on a neighbourhood-level socioeconomic index: 25.6% of E-Risk families live in “wealthy achiever” neighbourhoods compared to 25.3% nationwide; 5.3% vs. 11.6% live in “urban prosperity” neighbourhoods; 29.6% vs. 26.9% live in “comfortably off” neighbourhoods; 13.4% vs. 13.9% live in “moderate means” neighbourhoods, and 26.1% vs. 20.7% live in “hard-pressed” neighbourhoods [ACORN: A Classification of Residential Neighbourhoods, CACI Ltd.]. E-Risk underrepresents “urban prosperity” households because they are likely to be childless. **Figure S1** shows E-Risk families’ addresses are a near-perfect match to the deciles of the UK’s 2015 Lower-layer Super Output Area (LSOA) Index of Multiple Deprivation (IMD) which averages 1,500 residents; approximately 10% of the cohort fills each of IMD’s 10% bands for the UK.

### Missingness at age 18

There were no differences between those who did and did not take part at age 18 in terms of socioeconomic status (SES) assessed when the cohort was initially defined, $\chi2$(2, N=2,232)=0.86, *p*=0.65; age-5 IQ scores, t(2,208)=0.98, p=0.33; or age-5 emotional or behavioural problems, t(2,230)=0.40, *p*=0.69 and t(2,230)=0.41, *p*=0.68, respectively.

### Primary caregiver information

Where mothers were not available, other primary caregivers gave responses to interviewers that were recorded under the “mothers questionnaire”. Table S1 shows a summary of other caregivers that recorded these responses.

### Missing social isolation data in E-Risk

Cronbach alpha for the combined score was 0.68 at age 5, 0.73 at age 7, 0.75 at age 10 and 0.78 at age 12 (α for mother report at each age = 0.64-0.76, α for teacher report at each age = 0.68-0.76). Missingness was 0% at age 5, 2.42% at age 7, 4.21% at age 10 and 3.90% at age 12. At the individual level, 3.99% (N=89) of children were missing one assessment, 2.06% (N=46) of children were missing two and 0.81% (N=18) of children were missing the remaining three after the initial age 5 assessment. Social isolation data was assumed to be missing at random (MAR) as there were no significant differences between those who were missing data from three assessments and those who were not on IQ (t=1.22, *p*=0.23), internalising behaviours (t=-0.56, *p*=0.58) and externalising behaviours (t=0.36, *p*=0.72). There were also no differences in social isolation at age five (t=0.71, *p*=0.49) for individuals missing the remaining three time points and those who were not. Thus, all individuals were included in subsequent analyses (N=2,232). For the combined score, when the teacher report was missing, the mother report score was used as the combined score.


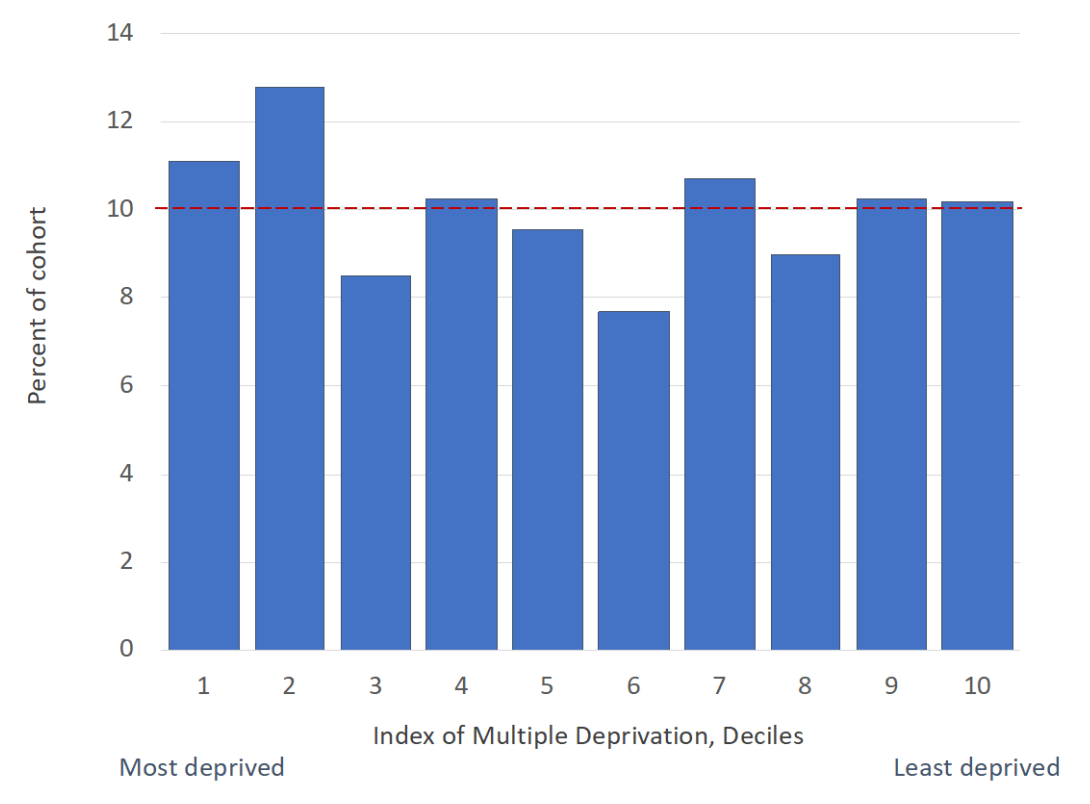


## Figure S1. Population representativeness of the E-Risk sample

Figure adapted from Reuben et al 2020 supplementary materials.^1^

| Table S1: percentage of primary caregivers that completed the “mother report” questionnaire | | | | | |
| --- | --- | --- | --- | --- | --- |
| N = 1,116 | Mother report | Mother and father combined | Father | Other | Missing |
| Age 5 | 98.7% | 0.4% | 0.7% | 0.2% | 0% |
| Age 7 | 96.3% | 0.1% | 1.0% | 0.2% | 2.4% |
| Age 10 | 93.8% | 0.0% | 1.6% | 0.4% | 4.2% |
| Age 12* | 93.0% | 0.0% | 2.2% | 0.9% | 3.9% |
| *The age 12 interview also consisted of an “other parent” questionnaire. | | | | | |

| Table S2: participants with complete data for social isolation and ADHD symptom sum scores across all time points | | |
| --- | --- | --- |
|  |  | **N with complete data (%)**  At age 5, 7, 10, and 12 |
| **Social isolation** | Mother report | 2075 (92.97%) |
|  | Teacher report | 1411 (62.27%) |
|  | Combined | 2079 (93.15%) |
| **ADHD symptoms** | Mother report | 2073 (92.88%) |
|  | Teacher report | 1342 (60.13) |
|  | Combined | 2078 (93.10%) |
| **Social isolation and ADHD symptoms** | Combined | 2078 (93.10%) |

# Study items

## Supplement 2

### ADHD diagnosis information

Meeting diagnostic criteria for ADHD required a score of six or more for inattention or hyperactivity/impulsivity reported by mothers or teachers in the past six months, and the other informant must have endorsed at least two symptoms. At age 5, 6.6% (n=138) of the full cohort met criteria for ADHD, 5.4% at age 7 (n=109), 3.5% at age 10 (n=72) and 3.4% at age 12 (n=68). In total, 247 participants (12.1%) met criteria for ADHD across childhood: 6.8% at age 5 (131/1921), 5.4% at age 7 (102/1880), 3.4% at age 10 (65/1912), and 3.4% at age 12 years (64/1884).

| Table S3. Pairwise correlations between social isolation and ADHD across ages 5, 7, 10, and 12 | | | | | | | | |
| --- | --- | --- | --- | --- | --- | --- | --- | --- |
|  | 1 | 2 | 3 | 4 | 5 | 6 | 7 | 8 |
| 1. ADHD age 5 | 1.00 |  |  |  |  |  |  |  |
| 2. Social isolation age 5 | 0.37 | 1.00 |  |  |  |  |  |  |
| 3. ADHD age 7 | 0.59 | 0.26 | 1.00 |  |  |  |  |  |
| 4. Social isolation age 7 | 0.30 | 0.49 | 0.37 | 1.00 |  |  |  |  |
| 5. ADHD age 10 | 0.48 | 0.18 | 0.56 | 0.27 | 1.00 |  |  |  |
| 6. Social isolation age 10 | 0.28 | 0.36 | 0.31 | 0.49 | 0.40 | 1.00 |  |  |
| 7. ADHD age 12 | 0.46 | 0.17 | 0.54 | 0.21 | 0.65 | 0.27 | 1.00 |  |
| 8. Social isolation age 12 | 0.30 | 0.32 | 0.31 | 0.41 | 0.33 | 0.58 | 0.39 | 1.00 |
| All entries are Pearson correlations. Pairwise complete estimation. ADHD = attention deficit hyperactivity disorder. All correlations significant at p<0.001. | | | | | | | | |

| Table S4. Full list of items used in the current study | | |
| --- | --- | --- |
| **Construct** | **Subscale** | **Item** |
| Social isolation | CBCL | Would rather be alone than with others |
|  |  | Not liked by other children |
|  |  | Doesn’t get along with other children |
|  |  | Feels or complains that no-one loves him/her |
|  |  | Withdrawn, doesn’t get involved with others |
|  |  | Complains of loneliness |
| ADHD | CBCL: Inattention | Can't concentrate, can't pay attention for long, short attention span |
|  |  | Fails to finish things he\she starts |
|  |  | Inattentive or easily distracted |
|  |  | Doesn't pay attention to details |
|  |  | Doesn't notice when people speak to him\her |
|  |  | Doesn't organise him\herself well |
|  |  | Doesn't like activities that require a lot of attention and effort |
|  |  | Loses things, like toys and clothes |
|  |  | Forgets what he\she is doing |
|  | CBCL: Hyperactivity/ impulsivity | Impulsive or acts without thinking |
|  |  | Interrupts conversations or games |
|  |  | Blurts out answers before the whole question has been asked |
|  |  | Has difficulty waiting for his\her turn |
|  |  | Fidgety or squirmy |
|  |  | Very restless, has difficulty staying seated for long |
|  |  | Cannot settle to anything for more than a few moments, quick |
|  |  | Seems to be "on the go" or acts as if "driven by a motor" |
|  |  | Talks too much |
| Antisocial behaviour | CBCL: Aggression | Argues a lot |
|  |  | Bragging, boasting |
|  |  | Bullying or threatening people |
|  |  | Demands a lot of attention |
|  |  | Destroys his/her own things |
|  |  | Destroys things belonging to family or others |
|  |  | Disobedient at home |
|  |  | Disobedient at school |
|  |  | Easily jealous |
|  |  | Gets in many fights |
|  |  | Physically attacks people |
|  |  | Screams a lot |
|  |  | Showing off or clowning |
|  |  | Stubborn or bad-tempered |
|  |  | Sudden changes in mood/mood swings |
|  |  | Talks too much |
|  |  | Teases a lot |
|  |  | Temper tantrums or hot temper |
|  |  | Unusually loud |
|  | CBCL: Delinquency | Doesn't seem to feel guilty after misbehaving |
|  |  | Hangs around with others who get in trouble |
|  |  | Lying or cheating |
|  |  | Prefers being with older children |
|  |  | Runs away from home |
|  |  | Sets fires |
|  |  | Steals at home |
|  |  | Steals outside the home |
|  |  | Swearing or bad language |
|  |  | Thinks about sex too much* |
|  |  | Skips school/truants |
|  |  | Uses drugs for non-medical purposes* |
|  |  | Drinks alcohol w/o parents’ approval* |
|  |  | Smokes tobacco* |
|  |  | Vandalism, damaging public property |
|  |  | Cruel to animals** |
|  |  | Cruel or nasty to other people** |
|  |  | Breaks rules at home/school/elsewhere** |
|  | Conduct disorder and Oppositional Defiant | Angry and hostile |
|  |  | Annoys people on purpose |
|  |  | Irritable/touchy/quick to fly off handle |
|  |  | Blames others for things |
|  |  | Spiteful, tries to get revenge |
|  |  | Hits others with things that could hurt |
|  |  | Take something from other child w/ force |
|  |  | Breaks into place where not allowed |
|  |  | Stays out past time should be home |
| Prosocial | Rutter Parent Questionnaire | Tries to stop quarrels or fights |
|  |  | Tries to be fair in games |
|  |  | Considerate of other people's feelings |
|  |  | Tries to help someone who has been hurt |
|  |  | Volunteers to help around the house or garden |
|  |  | Kind to younger children |
|  |  | Comforts a child who is crying or upset |
|  |  | Shares out treats with friends |
|  |  | Helps other children who are feeling ill |
|  |  | Kind to animals |
| *not included at age 5; ** additional items from Rutter’s Questionnaire | | |

#

# Conceptual longitudinal mediation


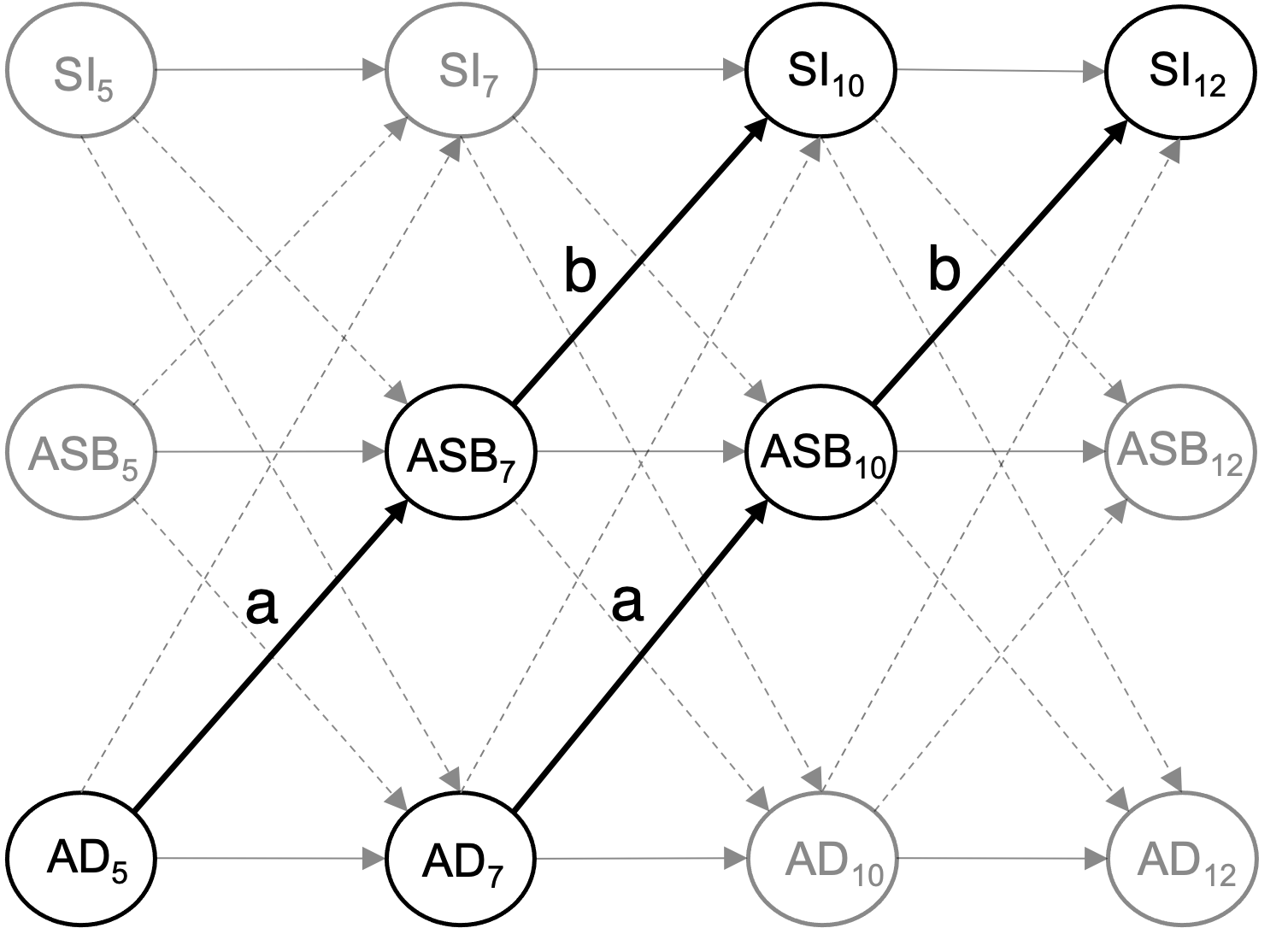


## Figure S2: Longitudinal mediation model using three-variable RI-CLPM

Random intercepts for ADHD, antisocial behaviour, and social isolation are not pictured here for clarity. AD: ADHD symptoms; ASB: antisocial behaviour; SI: social isolation. Longitudinal within-person cross-lag indirect mediation paths are indicted in bold and calculated a*b.

# Longitudinal measurement invariance

## Supplement 3

Measurement invariance was tested applying RI-CLPM in four steps:

1. The configural model - tests pattern of free and fixed loadings
2. Weak factorial invariance - constrain the factor loadings to be the same
3. Strong factorial invariance - equates the item intercepts
4. The latent RI-CLPM - factor loadings from items are used instead of sum scores

We conducted this separately for mother and teacher reports, and stratified by inattention, hyperactivity, and combined ADHD. Findings for each model and each step are summarised in the table below. Please go to [Github](https://github.com/knthompson26/social-isolation-ADHD-bidirectional-effects) for full analysis descriptions with code. All models assess bidirectional associations between social isolation and ADHD. As we have a large sample and many items, we will not rely on Chi square difference tests, instead a difference in fit (increase or decrease depending on statistic) of smaller than 0.01 was considered acceptable. Weak and strong invariance held for all models, whereas the latent model showed substantially worse fit. The latent model held for teacher report inattention.

| Table S5. Measurement invariance testing for RI-CLPM using social isolation and ADHD | | | | |
| --- | --- | --- | --- | --- |
| **Reporter** | **ADHD presentation** | **Step (model)** | **Overall fit** | **Difference in model fit**  *significantly worse |
| Mother | Inattention | 1 Configural | Good | NA (baseline) |
|  |  | 2 Weak | Good | Better fit |
|  |  | 3 Strong | Good | Same fit |
|  |  | 4 Latent | Poor | Worse fit***** |
| Mother | Hyperactivity | 1 Configural | Good | NA (baseline) |
|  |  | 2 Weak | Good | Worse fit |
|  |  | 3 Strong | Good | Same fit |
|  |  | 4 Latent | Poor | Worse fit***** |
| Teacher | Inattention | 1 Configural | Good | NA (baseline) |
|  |  | 2 Weak | Good | Worse fit |
|  |  | 3 Strong | Good | Same fit |
|  |  | 4 Latent | Good | Worse fit |
| Teacher | Hyperactivity | 1 Configural | Good | NA (baseline) |
|  |  | 2 Weak | Good | Worse fit |
|  |  | 3 Strong | Good | Same fit |
|  |  | 4 Latent | Poor | Worse fit* |

# Constraint testing for CLPM

## Supplement 4

To test if associations between social isolation and ADHD symptoms were consistent over time, we constrained the cross-lag effects to be equal across each lag (5-7, 7-10, 10-12). We tested if this equality constraint led to a significant decrease in model fit. If non-significant, cross-lag associations were consistent across childhood.

| Table S6. Equality constraint testing for combined report CLPM: 𝝌² difference test statistics | | | |
| --- | --- | --- | --- |
|  | **𝝌²**$\Delta$ | **df** $\Delta$ | ***p*** |
| **ADHD** | | | |
| Non-constrained (Baseline) | 408.34 |  |  |
| Constrained both cross-lags | 2.54 | 4 | 0.632 |
| **Hyperactivity** | | | |
| Non-constrained (Baseline) | 366.12 |  |  |
| Constrained both cross-lags | 4.67 | 4 | 0.322 |
| **Inattention** | | | |
| Non-constrained (Baseline) | 427.82 |  |  |
| Constrained both cross-lags | 1.68 | 4 | 0.795 |
| $\chi$²$\Delta$: Difference in Chi Square Statistic; df$\Delta$: difference in degrees of freedom. | | | |

# Variances and covariances of random intercepts from RI-CLPM

| Table S7. Variances and covariances of the random intercepts for the total ADHD and social isolation RI-CLPM | | |
| --- | --- | --- |
|  | ADHD RI | Isolation RI |
| ADHD RI | 3.206 (1.000) |  |
| Isolation RI | 0.661 (0.540) | 0.467 (1.000) |
| Standardised estimates in parentheses. RI = random intercept. All significant at *p*<0.001. At the between-person level, both random intercepts (isolation and ADHD) had significant variance. This shows that there are stable, trait-like differences between individuals for social isolation and ADHD. | | |

| Table S8. Variances and covariances of the random intercepts for the total ADHD, social isolation and antisocial behaviour RI-CLPM | | | |
| --- | --- | --- | --- |
|  | ADHD RI | Isolation RI | Antisocial RI |
| ADHD RI | 3.149 (1.000) |  |  |
| Isolation RI | 0.657 (0.541) | 0.469 (1.000) |  |
| Antisocial RI | 9.498 (0.789) | 2.821 (0.607) | 46.063 (1.000) |
| Standardised estimates in parentheses. RI = random intercept. All significant at *p*<0.001 | | | |

| Table S9. Variances and covariances of the random intercepts for the total ADHD, social isolation and prosocial behaviour RI-CLPM | | | |
| --- | --- | --- | --- |
|  | ADHD RI | Isolation RI | Prosocial RI |
| ADHD RI | 3.194 (1.000) |  |  |
| Isolation RI | 0.661 (0.538) | 0.472 (1.000) |  |
| Prosocial RI | -1.571 (-0.494) | -0.653 (-0.534) | 3.169 (1.000) |
| Standardised estimates in parentheses. RI = random intercept. All significant at *p*<0.001 | | | |

# Constraint testing for RI-CLPM

## Supplement 5

To test if associations between social isolation and ADHD symptoms were consistent over time, we constrained the autoregressive and cross-lag effects to be equal across each lag (5-7, 7-10, 10-12). We tested if this equality constraint led to a significant decrease in model fit. If non-significant, associations were consistent across childhood. When a model did not show significant decrease in model fit, we then used this as the new baseline. For example, the constraining cross-lag of isolation to ADHD was compared to the previous non-significant cross-lag constraint of ADHD to isolation. For the combined reporter models, all cross-lags were constrained over time with no loss in fit. For the mother report, all cross-lags were constrained over time with no loss in fit. For the teacher report, all autoregressive and cross-lag effects could be constrained to be equal over time.

| Table S10. Equality constraint testing for combined report RI-CLPM: 𝝌² difference test statistics | | | |
| --- | --- | --- | --- |
|  | **𝝌²**$\Delta$ | **df** $\Delta$ | **p** |
| **ADHD** | | | |
| Baseline RI-CLPM | 70.82 |  |  |
| Constraining only autoregressive lag in ADHD | 9.02 | 2 | 0.011 |
| Constraining only autoregressive lag in social isolation | 11.56 | 2 | 0.003 |
| Constraining cross lag ADHD predicts social isolation | 0.49 | 2 | 0.783 |
| Constraining cross lag social isolation predicts ADHD | 1.41 | 2 | 0.495 |
| **Hyperactivity** | | | |
| Baseline RI-CLPM | 54.87 |  |  |
| Constraining only autoregressive lag in hyperactivity | 10.1 | 2 | 0.006 |
| Constraining only autoregressive lag in social isolation | 11.28 | 2 | 0.003 |
| Constraining cross lag hyperactivity predicts social isolation | 0.34 | 2 | 0.84 |
| Constraining cross lag social isolation predicts hyperactivity | 1.44 | 2 | 0.487 |
| **Inattention** | | | |
| Baseline RI-CLPM | 66.13 |  |  |
| Constraining only autoregressive lag in inattention | 9.76 | 2 | 0.007 |
| Constraining only autoregressive lag in social isolation | 12.22 | 2 | 0.002 |
| Constraining cross lag inattention predicts social isolation | 1.32 | 2 | 0.517 |
| Constraining cross lag social isolation predicts inattention | 0.91 | 2 | 0.635 |
| $\chi$²$\Delta$: Difference in Chi Square Statistic; df$\Delta$: difference in degrees of freedom. | | | |

| Table S11. Equality constraint testing for mother report RI-CLPM: 𝝌² difference test statistics | | | |
| --- | --- | --- | --- |
|  | **𝝌²**$\Delta$ | **df** $\Delta$ | **p** |
| **ADHD** | | | |
| Baseline RI-CLPM | 95.49 |  |  |
| Constraining only autoregressive lag in ADHD | 21.51 | 2 | <0.001 |
| Constraining only autoregressive lag in social isolation | 12.99 | 2 | 0.001 |
| Constraining cross lag ADHD predicts social isolation | 0.08 | 2 | 0.961 |
| Constraining cross lag social isolation predicts ADHD | 1.72 | 2 | 0.422 |
| **Hyperactivity** | | | |
| Baseline RI-CLPM | 76.72 |  |  |
| Constraining only autoregressive lag in hyperactivity | 24.17 | 2 | <0.001 |
| Constraining only autoregressive lag in social isolation | 13.12 | 2 | 0.001 |
| Constraining cross lag hyperactivity predicts social isolation | 0.12 | 2 | 0.941 |
| Constraining cross lag social isolation predicts hyperactivity | 1.87 | 2 | 0.391 |
| **Inattention** | | | |
| Baseline RI-CLPM | 96.68 |  |  |
| Constraining only autoregressive lag in inattention | 20.76 | 2 | <0.001 |
| Constraining only autoregressive lag in social isolation | 13.65 | 2 | 0.001 |
| Constraining cross lag inattention predicts social isolation | 0.32 | 2 | 0.848 |
| Constraining cross lag social isolation predicts inattention | 0.29 | 2 | 0.862 |
| $\chi$²$\Delta$: Difference in Chi Square Statistic; df$\Delta$: difference in degrees of freedom. | | | |

| Table S12. Equality constraint testing for teacher report RI-CLPM: 𝝌² difference test statistics | | | |
| --- | --- | --- | --- |
|  | **𝝌²**$\Delta$ | **df** $\Delta$ | **p** |
| **ADHD** | | | |
| Baseline RI-CLPM | 49.48 |  |  |
| Constraining autoregressive and cross-lags | 4.92 | 8 | 0.766 |
| **Hyperactivity** | | | |
| Baseline RI-CLPM | 50.62 |  |  |
| Constraining autoregressive and cross-lags | 8.64 | 8 | 0.373 |
| **Inattention** | | | |
| Baseline RI-CLPM | 43.71 |  |  |
| Constraining autoregressive and cross-lags | 6.28 | 8 | 0.615 |
| $\chi$²$\Delta$: Difference in Chi Square Statistic; df$\Delta$: difference in degrees of freedom. | | | |

# Do prosocial or antisocial behaviours mediate the association between ADHD and isolation?

## Supplement 6

CLPM mediation models showed adequate fit (antisocial: CFI=0.96, TLI=0.94, RMSEA=0.08, SRMR=0.06; prosocial: CFI=0.94, TLI=0.89, RMSEA=0.08, SRMR=0.06). Prosocial behaviours partially mediated the effect of ADHD on social isolation (a*b indirect mediation β=0.005, *p*<0.001) and the effect of isolation on ADHD (a*b β=0.006, *p*<0.001). Antisocial behaviours partially mediated the effect of ADHD on social isolation (a*b β=0.007, *p*<0.001), but not the effect of isolation on ADHD (a*b β=-0.002, *p*=0.380).

RI-CLPM mediation models showed great fit (antisocial: CFI=0.99, TLI=0.99, RMSEA=0.03, SRMR=0.03; prosocial: CFI=0.99, TLI=0.99, RMSEA=0.03, SRMR=0.03). Path diagrams for RI-CLPM mediation models are shown in **Figure S2**, random intercepts have been removed from the figure for clarity. In contrast to the CLPM, no significant a*b indirect mediation paths were identified. Rather than a mediating effect, antisocial behaviours appeared to confound the association between ADHD and social isolation. When antisocial behaviours were added to the RI-CLPM, the effect of ADHD on isolation was no longer significant. Instead, antisocial behaviours independently predicted both social isolation and ADHD behaviours (**Figure S2, panel A**). Prosocial behaviours also did not provide a mediating effect. Rather, we found that increased prosocial behaviours at age 5 predicted decreased ADHD symptoms at age 7 which in turn predicted decreased social isolation at age 12 (**Figure S2, panel B**). CLPM and RI-CLPM cross-lag path estimates can be found in **Table S12**.

**
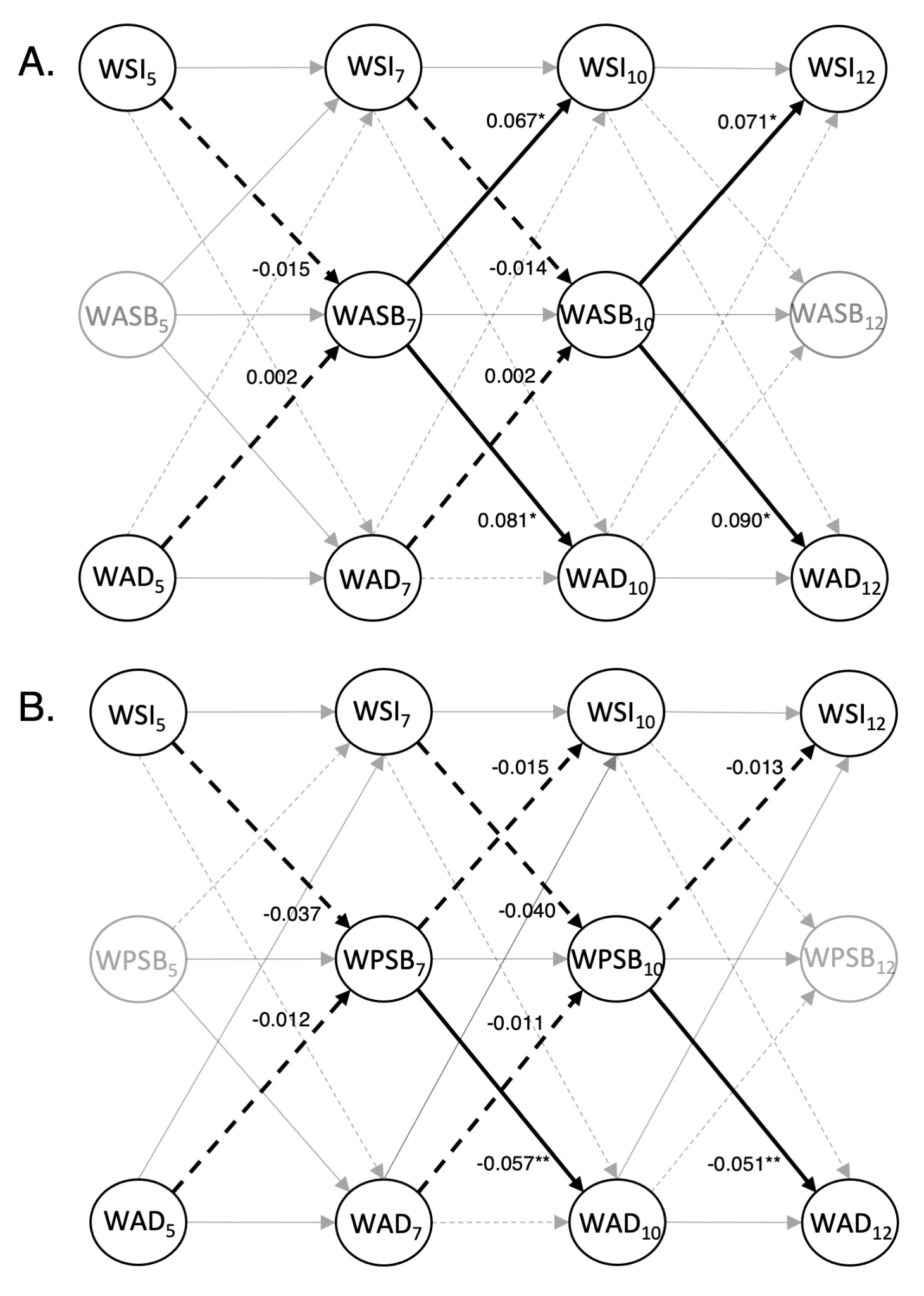
**

## Figure S3. Longitudinal medication model

Panel A shows the RI-CLPM representing the longitudinal mediating effect of antisocial behaviours on the association between ADHD and social isolation across ages 5, 7, 10, and 12. Panel B shows the RI-CLPM representing the longitudinal mediating effect of prosocial behaviours on the association between ADHD and social isolation across ages 5, 7, 10, and 12. WSI: within-person level factor of social isolation; WAD: within-person level factor of ADHD; WASB: within-person level factor of antisocial behaviours; WPSB: within-person level factor of prosocial behaviours. Non-significant paths are indicated by dashed lines. Significant paths are indicated by solid lines. Meditation paths are in bold. Time point of assessment is indicated in the subscript. Combined mother and teacher scores used at all time points. Both models have cross-lag paths constrained to be equal across time.

| Table S13. Mediation random-intercept cross-lagged panel model for how prosocial and antisocial behaviour mediate the association between ADHD and social isolation. | | | |
| --- | --- | --- | --- |
|  |  | **Indirect effect from ADHD to isolation**  **(Standardised β)** | **Indirect effect from isolation to ADHD**  **(Standardised β)** |
| **CLPM** | | | |
| **Prosocial behaviour** | | | |
|  | ADHD | 0.005*** | 0.006*** |
|  | Hyperactivity | 0.005*** | 0.006*** |
|  | Inattention | 0.005*** | 0.008*** |
| **Antisocial behaviour** | | | |
|  | ADHD | 0.007*** | -0.002 |
|  | Hyperactivity | 0.007*** | -0.001 |
|  | Inattention | 0.006** | -0.002 |
| **RI-CLPM** | | | |
| **Prosocial behaviour** | | | |
|  | ADHD | 0.000 | 0.002 |
|  | Hyperactivity | 0.000 | 0.002 |
|  | Inattention | 0.000 | 0.002 |
| **Antisocial behaviour** | | | |
|  | ADHD | 0.000 | 0.001 |
|  | Hyperactivity | 0.002 | 0.000 |
|  | Inattention | 0.000, -0.006 | -0.001, 0.011 |
| Significance indicated by: ~p=0.05, *p<0.05, **p<0.01, ***p<0.001. All RI-CLPM mediation models use combined (averaged) mother and parent reports. Mediation effects are calculated a*b (e.g. effect of ADHD to antisocial behaviour*effect of antisocial behaviour to social isolation). Apart from RI-CLPM antisocial inattention, all models constrained cross-lag effects to be equal over time. RI-CLPM antisocial inattention model cross lags were freed to differ over time: β notation is given as, age 5-7, age 7-10. | | | |

# Sex and SES differences for mother and teacher report models.

| Table S14. Random-intercept cross-lagged panel model (RI-CLPM) fit statistics for combined ADHD symptoms, hyperactivity, and inattention, split by reporter | | | | | | | | |
| --- | --- | --- | --- | --- | --- | --- | --- | --- |
|  | **Scaled** $\chi$**²** | **df** | **CFI** | **TLI** | **Adjusted BIC** | **RMSEA** | **RMSEA 95% CI** | **SRMR** |
| **Mother report** | | | | | | | | |
| ADHD | 59.77 | 13 | 0.99 | 0.98 | 72912.94 | 0.05 | 0.04,0.07 | 0.03 |
| Hyperactivity | 52.35 | 13 | 0.99 | 0.98 | 64296.44 | 0.05 | 0.03,0.06 | 0.03 |
| Inattention | 57.16 | 13 | 0.99 | 0.97 | 61996.43 | 0.05 | 0.04,0.06 | 0.03 |
| **Teacher report** | | | | | | | | |
| ADHD | 26.05 | 17 | 0.99 | 0.98 | 59984.86 | 0.02 | NA,0.04 | 0.03 |
| Hyperactivity | 31.38 | 17 | 0.98 | 0.97 | 50813.85 | 0.03 | 0.01,0.05 | 0.03 |
| Inattention | 24.15 | 17 | 0.99 | 0.99 | 51845.20 | 0.02 | NA,0.04 | 0.03 |
| $\chi$²: Chi Square Statistic; CFI: Comparative Fit Index; TLI: Tucker-Lewis Index; BIC: Bayesian Information Criterion RMSEA: Root Mean Square Error of Approximation, CI: Confidence Interval; SRMR: Standardised Root Mean Squared Residual; ADHD: Attention Deficit Hyperactivity Disorder. All fit statistics reported are robust. Mother report RI-CLPM have cross-lagged paths constrained to be equal across time. Teacher report RI-CLPM have autoregressive and cross-lagged paths constrained to be equal across time. | | | | | | | | |

| Table S15. Random-intercept cross-lagged panel model (RI-CLPM) cross-lag effect estimates stratified by sex, socioeconomic status (SES), for each informant (mother and teacher) | | | | | | | | |
| --- | --- | --- | --- | --- | --- | --- | --- | --- |
|  |  | **ADHD predicting isolation**  **(Standardised β)** | | | **Isolation predicting ADHD**  **(Standardised β)** | | | **𝝌² Difference test** |
|  |  | Age 5-7 | Age 7-10 | Age 10-12 | Age 5-7 | Age 7-10 | Age 10-12 |  |
| **Mother report** | | | | | | | | |
| **ADHD sex differences** | | | | | | | | 𝝌²$\Delta$=5.56 , df$\Delta$=8 , *p*=0.695 |
|  | Female | 0.045 | 0.033 | 0.026 | 0.013 | 0.018 | 0.019 |  |
|  | Male | 0.086 | 0.064 | 0.056 | 0.051 | 0.054 | 0.063 |  |
| **Hyperactivity sex differences** | | | | | | | | 𝝌²$\Delta$=2.63 , df$\Delta$=8 , *p*=0.955 |
|  | Female | 0.040 | 0.029 | 0.021 | 0.026 | 0.039 | 0.042 |  |
|  | Male | 0.080 | 0.061 | 0.049 | 0.035 | 0.041 | 0.051 |  |
| **Inattention sex differences** | | | | | | | | 𝝌²$\Delta$=9.99 , df$\Delta$=8 , *p*=0.265 |
|  | Female | 0.045 | 0.035 | 0.031 | 0.000 | 0.000 | 0.000 |  |
|  | Male | 0.072 | 0.052 | 0.052 | 0.065 | 0.061 | 0.069 |  |
| **ADHD SES differences** | | | | | | | | 𝝌²$\Delta$=14.31 , df$\Delta$=16 , *p*=0.575 |
|  | Low | 0.143* | 0.110* | 0.095* | 0.049 | 0.055 | 0.065 |  |
|  | Middle | 0.052 | 0.040 | 0.032 | 0.002 | 0.002 | 0.002 |  |
|  | High | 0.013 | 0.009 | 0.008 | 0.040 | 0.049 | 0.057 |  |
| **Hyperactivity SES differences** | | | | | | | | 𝝌²$\Delta$=13.81 , df$\Delta$=16 , *p*=0.612 |
|  | Low | 0.093 | 0.067 | 0.059 | 0.040 | 0.045 | 0.054 |  |
|  | Middle | 0.078 | 0.067 | 0.048 | 0.021 | 0.030 | 0.033 |  |
|  | High | 0.014 | 0.010 | 0.008 | 0.021 | 0.031 | 0.038 |  |
| **Inattention SES differences** | | | | | | | | 𝝌²$\Delta$=16.35 , df$\Delta$=16 , *p*=0.428 |
|  | Low | 0.160** | 0.128** | 0.114** | 0.060 | 0.066 | 0.076 |  |
|  | Middle | 0.002 | 0.002 | 0.002 | -0.035 | -0.038 | -0.037 |  |
|  | High | 0.013 | 0.010 | 0.011 | 0.059 | 0.057 | 0.064 |  |
| **Teacher report** | | | | | | | | |
| **ADHD sex differences** | | | | | | | | 𝝌²$\Delta$=2.84, df$\Delta$=4 , *p*=0.583 |
|  | Female | 0.019 | 0.013 | 0.011 | -0.067 | -0.089 | -0.0111 |  |
|  | Male | 0.133** | 0.123** | 0.105** | 0.005 | 0.007 | 0.008 |  |
| **Hyperactivity sex differences** | | | | | | | | 𝝌²$\Delta$=4.47 , df$\Delta$=4 , *p*=0.345 |
|  | Female | 0.052 | 0.035 | 0.028 | -0.054 | -0.072 | -0.078 |  |
|  | Male | 0.129** | 0.117** | 0.098** | 0.023 | 0.029 | 0.035 |  |
| **Inattention sex differences** | | | | | | | | 𝝌²$\Delta$=3.47 , df$\Delta$=4 , *p*=0.481 |
|  | Female | -0.014 | -0.010 | -0.008 | -0.063 | -0.082 | -0.010 |  |
|  | Male | 0.103* | 0.098* | 0.089* | -0.000 | -0.000 | -0.001 |  |
| **ADHD SES differences** | | | | | | | | 𝝌²$\Delta$=8.42, df$\Delta$=8 , *p*=0.393 |
|  | Low | 0.119~ | 0.114~ | 0.089~ | -0.023 | 0.029 | -0.030 |  |
|  | Middle | 0.099* | 0.084* | 0.070* | -0.038 | -0.054 | -0.067 |  |
|  | High | 0.013 | 0.010 | 0.010 | -0.025 | -0.028 | -0.046 |  |
| **Hyperactivity SES differences** | | | | | | | | 𝝌²$\Delta$=9.48 , df$\Delta$=8 , *p*=0.303 |
|  | Low | 0.155** | 0.153** | 0.126** | 0.021 | 0.025 | 0.027 |  |
|  | Middle | 0.128* | 0.103* | 0.079* | -0.024 | -0.036 | -0.040 |  |
|  | High | -0.012 | -0.009 | -0.008 | -0.027 | -0.033 | -0.047 |  |
| **Inattention SES differences** | | | | | | | | 𝝌²$\Delta$=6.74 , df$\Delta$=8 , *p*=0.563 |
|  | Low | 0.065 | 0.058 | 0.046 | -0.039 | -0.048 | -0.049 |  |
|  | Middle | 0.048 | 0.047 | 0.041 | -0.044 | -0.058 | -0.074 |  |
|  | High | 0.025 | 0.021 | 0.023 | -0.015 | -0.016 | -0.028 |  |
| Significance indicated by: ~p=0.05, *p<0.05, **p<0.01, ***p<0.001. $\chi$²: Chi Square Statistic. Sex and SES models use combined (averaged) reports from parents and teachers. Robust 𝝌² used in all models. 𝝌² difference test is not applicable for reporter differences as model created from separate data (not nested). Combined report and mother report include constrained cross-lag effects. Teacher report models include constrained autoregressive and cross-lag effects. $\Delta$ = difference. | | | | | | | | |

| Table S16. Iterations of models using different combinations of reporters for isolation and ADHD | | | | |
| --- | --- | --- | --- | --- |
| **Model name** | **Social isolation** | **ADHD** | **ADHD predicts social isolation** | **Social isolation predicts ADHD** |
| **ADHD** | | | | |
| RICLPM | Mother report | Mother report | No | No |
| RICLPMt | Teacher report | Teacher report | Yes | No |
| RICLPMcomb | Combined report | Combined report | Yes | No |
| RICLPMadt | Combined report | Teacher report | No | No |
| RICLPMadm | Combined report | Mother report | Yes | Yes |
| RICLPMsit | Teacher report | Combined report | Yes | No |
| RICLPMsim | Mother report | Combined report | Yes | No |
| **Hyperactivity** | | | | |
| RICLPM_hyp | Mother report | Mother report | No | No |
| RICLPMt_hyp | Teacher report | Teacher report | Yes | No |
| RICLPMcomb_hyp | Combined report | Combined report | Yes | No |
| RICLPMadt_hyp | Combined report | Teacher report | No | No |
| RICLPMadm_hyp | Combined report | Mother report | Yes | Yes |
| RICLPMsit_hyp | Teacher report | Combined report | Yes | No |
| RICLPMsim_hyp | Mother report | Combined report | No | No |
| **Inattention** | | | | |
| RICLPM_inat | Mother report | Mother report | No | No |
| RICLPMt_inat | Teacher report | Teacher report | No | No |
| RICLPMcomb_inat | Combined report | Combined report | No | No |
| RICLPMadt_inat | Combined report | Teacher report | No | No |
| RICLPMadm_inat | Combined report | Mother report | No | Yes |
| RICLPMsit_inat | Teacher report | Combined report | No | No |
| RICLPMsim_inat | Mother report | Combined report | Yes | No |
| When assessing many different iterations of the models using different combinations of informants, the association appears to persist when teachers are assessing social isolation in the classroom. | | | | |

# References

1. Reuben A, Sugden K, Arseneault L, et al. Association of Neighborhood Disadvantage in Childhood With DNA Methylation in Young Adulthood. *JAMA Netw Open*. 2020;3(6):e206095-e206095. doi:10.1001/jamanetworkopen.2020.6095
